# Normalized affective responsiveness following deep brain stimulation of the medial forebrain bundle in depression

**DOI:** 10.1038/s41398-023-02712-y

**Published:** 2024-01-08

**Authors:** Hannah Marlene Kilian, Bastian Schiller, Dora Margarete Meyer-Doll, Markus Heinrichs, Thomas Eduard Schläpfer

**Affiliations:** 1 Division of Interventional Biological Psychiatry, Department of Psychiatry and Psychotherapy Medical Center - University of Freiburg, Faculty of Medicine, DE-79104 Freiburg, Germany; 2 Department of Psychology, Laboratory for Biological Psychology, Clinical Psychology and Psychotherapy, University of Freiburg, DE-79104 Freiburg, Germany

**Keywords:** Human behaviour, Depression

## Abstract

Deep brain stimulation (DBS) of the supero-lateral medial forebrain bundle (slMFB) is associated with rapid and sustained antidepressant effects in treatment-resistant depression (TRD). Beyond that, improvements in social functioning have been reported. However, it is unclear whether social skills, the basis of successful social functioning, are systematically altered following slMFB DBS. Therefore, the current study investigated specific social skills (affective empathy, compassion, and theory of mind) in patients with TRD undergoing slMFB DBS in comparison to healthy subjects. 12 patients with TRD and 12 age- and gender-matched healthy subjects (5 females) performed the EmpaToM, a video-based naturalistic paradigm differentiating between affective empathy, compassion, and theory of mind. Patients were assessed before and three months after DBS onset and compared to an age- and gender-matched sample of healthy controls. All data were analyzed using non-parametric Mann-Whitney U tests. DBS treatment significantly affected patients’ affective responsiveness towards emotional versus neutral situations (i.e. affective empathy): While their affective responsiveness was reduced compared to healthy subjects at baseline, they showed normalized affective responsiveness three months after slMFB DBS onset. No effects occurred in other domains with persisting deficits in compassion and intact socio-cognitive skills. Active slMFB DBS resulted in a normalized affective responsiveness in patients with TRD. This specific effect might represent one factor supporting the resumption of social activities after recovery from chronic depression. Considering the small size of this unique sample as well as the explorative nature of this study, future studies are needed to investigate the robustness of these effects.

## Introduction

Approximately 30% of patients with depression do not respond to conventional treatment methods such as psychotherapy and pharmacotherapy [1]. Resistance to antidepressant treatments is associated with a reduced quality of life for the patients [2, 3]. Currently, deep brain stimulation (DBS) is under investigation as new emerging treatment method in psychiatry [4–7]. DBS is an invasive, non-lesional and highly focal treatment method that involves the bilateral implantation of electrodes into a selected brain area as well as the constant application of electrical impulses to this brain target. Electrical current is delivered from a pulse generator implanted subcutaneously in the region of the clavicular [8]. The supero-lateral medial forebrain bundle (slMFB) represents one of the brain targets for DBS electrode placement currently investigated in treatment-resistant depression (TRD) [9, 10]. DBS of the slMFB has shown promising results in terms of rapid and sustained antidepressant effects [11–15]. Beyond that, patients subjectively reported social functioning improvements [13, 16]. Considering that normal social functioning is crucial for a good quality of life [17, 18], reduces the mortality risk [19] and further plays an important role in the long-term stabilization after chronic diseases [20], it can be considered an important therapeutic outcome [9]. However, slMFB DBS effects beyond symptom improvement are rarely studied [21, 22] and the mechanisms of slMFB DBS improving poor social functioning in depression are unknown, so far [20]. Thus, the current study systematically investigates DBS treatment effects on social skills, the basis of successful social functioning [23]. Specifically, three higher-order social skills termed affective empathy, compassion, and theory of mind (ToM) are being investigated. Affective empathy, compassion, and ToM were assessed behaviorally before and three months after the onset of active slMFB DBS in patients with TRD and compared to a sample of age- and gender-matched healthy controls.

Affective empathy is defined as the ability to share positive and negative feelings of a counterpart [24]. Feelings of affective empathy might further induce positive feelings of warmth and care including the motivation to help another person and reduce their suffering (compassion/ concern). Study evidence shows that patients with depressive symptoms feel less affective empathy and compassion [20, 25, 26]. On the other side, feelings of affective empathy might also cause aversive feelings of stress [27], which seem to be prominent in patients with depression [25, 28–30]. Theory of Mind (ToM), also known as perspective-taking or mentalizing, describes the ability to understand and infer mental states of another person [31]. Data from studies investigating ToM in depression have been inconsistent [20, 28]. Meta-analyses have yielded impaired ToM skills [32, 33], while recent single studies revealed intact ToM skills either from self-report questionnaires or assessed with new naturalistic paradigms [25, 26, 30].

Considering the role social skill deficits play in the development, maintenance and re-occurrence of depressive symptoms [34–36], improving these deficits represents an important outcome in the antidepressant treatment. Pharmacotherapy as well as specifically developed psychotherapy (e.g. cognitive behavioral system of psychotherapy (CBASP) [37]; interpersonal therapy (IPT) [38]) have an impact on social skill deficits in depression, but effects are only small to moderate [20, 39, 40]. DBS of the slMFB, however, might directly influence social skills. This idea is based on neuroimaging data demonstrating that the slMFB as a connecting structure of brain regions of the mesolimbic pathway not only induces brain metabolism changes in the stimulated area but also distal to the stimulated target [41–43]. Importantly, neuronal regions associated with affective empathy, compassion and ToM partly overlap with these regions, e.g. the medial prefrontal cortex, the ventral tegmental area and the ventral striatum [24, 44–52]. It thus seems plausible to assume that slMFB DBS modulates social skill deficits associated with depression.

In this study, we aim to illuminate social functioning changes following slMFB DBS by systematically investigating behaviorally assessed affective empathy, compassion, and ToM before and after the onset of stimulation in a unique sample of patients with TRD. In order to do so, we used a naturalistic test paradigm based on video stimuli, the EmpaToM [24]. The EmpaToM has previously been validated using an established empathy task for behavioural outcomes as well as on a neuronal level by comparison of activation clusters with previous findings of meta-analyses [24]. Furthermore, this paradigm has been shown to significantly differentiate between affective empathy, compassion and ToM in studies with different (patient) samples [53–55]. Patients with TRD (*n* = 12) performed the EmpaToM both before the neurosurgical procedure with implantation of the DBS system and three months after the onset of active slMFB DBS. These data were then compared to an age- and gender-matched sample of healthy control subjects (HC) (*n* = 12). Based on reports of subjectively improved social functioning after slMFB DBS [26] and the neuronal overlap of regions stimulated by slMFB DBS and associated with social skills, we hypothesized that DBS normalizes impaired social skills in patients with TRD.

## Materials, subjects and methods

### Sample description and recruitment

Patients were recruited through the outpatient clinic of the Division of Interventional Biological Psychiatry, Department of Psychiatry and Psychotherapy, Medical Center, University of Freiburg. Inclusion criteria were a primary diagnosis of major depressive disorder, a current chronic episode ( > two years) or at least four previous episodes of depression, a minimum score of 21 of the Hamilton Depression Rating Scale (HDRS) [56] and a score of less than 45 in the Global Assessment of Functioning (GAF) [57]. All patients included were diagnosed with unipolar depression. Treatment-resistant depression (TRD) was defined as a lacking or inadequate response to all these treatments: (1) three different classes of antidepressants, (2) augmentation/combination therapy of primary antidepressants with other agents, (3) electroconvulsive therapy ( > 6 session) and (4) individual psychotherapy ( > 20 h). Adequacy of previous treatments was assessed with the Antidepressive Treatment History Form (ATHF) [58]. Furthermore, patients with a diagnosis of non-affective psychotic disorder, neurological disorder or medical illness affecting brain function, current or unstably remitted substance abuse, severe personality disorder and acute suicidal ideation were excluded (for a detailed descripition of inclusion and exclusion criteria see clinicaltrials.gov (NCT03653858) or previous publications, e.g. [11]). Data of 12 patients with TRD were analyzed and compared to 12 age- and gender-matched HC. The baseline data before DBS surgery (*n* = 21) have previously been published [26] and the patients reported in this study represent a subsample (*n* = 15) of participants who underwent surgery. Of this subsample, three patients did not take part in the follow-up measurement, resulting in a final sample of 12 patients. Healthy control subjects completed an online questionnaire and were eligible if they had no history of neurological or psychiatric disorders, no previous or current psychiatric or psychotherapeutic treatment and no current alcohol or drug abuse, as well as no current depressive symptoms (BDI < 10). All participants were fluent in German.

### Procedure

**Fig. 1:**
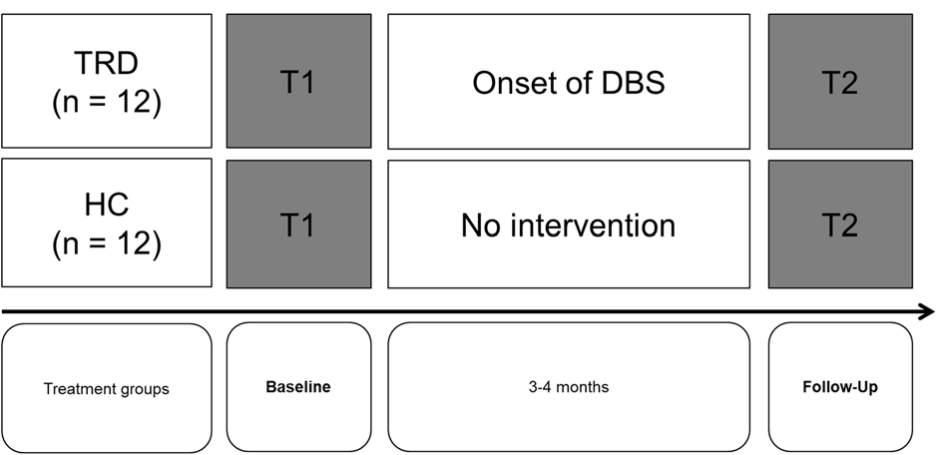
Study Procedure. Socio-affective and socio-cognitive skills were assessed in a sample of 12 patients with TRD (5 females) before and after three months of slMFB DBS (gray boxes) and compared to social skills of 12 age- and gender-matched healthy control subjects (HC).

Patients with TRD were tested two to four weeks before stereotactic surgery was performed (see Fig. 1). Stereotactic surgery contains the bilateral implantation of DBS electrodes in the selected brain target (slMFB) under local anesthesia as well as the implantation of the pulse generator in the region of the clavicular under general anesthesia (for a detailed description of the surgery procedure see [59]). The slMFB has been proposed as DBS target in depression considering its central location, interconnections with other DBS targets in depression (e.g. ventral striatum), and its association with reward and motivation seeking behavior [9, 10].

Follow-up data of patients with TRD were analyzed three months after the stimulation onset of slMFB DBS (active DBS). Patients were asked not to change pharmacotherapy or psychotherapy during the study trial. The assessment in the healthy subject sample was repeated three to four months after the first measurement (without intervention). This study was registered at the ‘Deutsches Register Klinischer Studien (DRKS)‘ (identifier DRKS00019092). Patients were recruited from the ongoing FORESEE III trial (clinicaltrials.gov with identifier: NCT03653858). The authors assert that all procedures contributing to this work comply with the ethical standards of the relevant national and institutional committees on human experimentation (affirmative vote of the University of Freiburgs’s Ethics Committee on 12/21/2017) and with the Helsinki Declaration of 1975, as revised in 2008. Written informed consents were signed by all participants before study participation.

### Measures

#### Clinical symptoms

Severity of symptoms of depression was assessed using self-report (*Beck Depression Inventory* (BDI), Hautzinger et al. [60]) as well as expert-rating instruments (*Montgomery-Åsberg Depression Rating Scale* (MADRS), Montgomery & Åsberg [61]; *Hamilton Depression Rating Scale* (HDRS), Hamilton [56]). These scales have reached the status of a gold standard for the evaluation of symptoms of depression according to the diagnostic criteria [62].

#### Social skills

The *EmpaToM* [24] is a video-based naturalistic test paradigm. In total, 48 short videos ( ~ 15 s) are presented that display people talking about a situation with either neutral or emotional (negative) valence (24 videos per condition) (for exemplary video stories see [24]). While neutral videos represent the control condition, videos with emotionally negative content represent the experimental condition. To measure affective empathy, participants are asked to rate their own current feelings (‚How do you feel?‘) on a dimensional scale ranging from ‚negative‘ (-2) to ‚positive‘ (2) after each video. The affective responsiveness is calculated as a difference score of empathic responses to emotional and neutral videos describing the ability to affectively resonate with others in response to different situations. For compassion, another question (‚How much compassion do you feel?‘) has to be answered on a dimensional scale ranging from ‚none‘ (0) to ‚very much‘ (6). Theory of mind is assessed by a multiple choice question referring to the thoughts of the person in the video (e.g. ‚Anna thinks that…‘). Participants have to select one out of three options and the accuracy (correct answers/total number of videos; min = 0, max = 1) is calculated. To control for attention and concentration abilities, half of the multiple choice questions demand factual reasoning skills (‚It is correct that…‘). The test thus comprises four conditions (12 trials per condition) with two video categories (neutral and emotional) and two task categories (ToM and non-ToM) (1: neutral, non-ToM; 2: emotional, non-ToM; 3: neutral, ToM; 4: emotional, ToM) (for a detailed description see [24]). For the main analyses, we combined the four categories so that only neutral and emotional or ToM and non-ToM were compared. The videos are presented in a different order for each participant and parallelized test versions presenting new videos were utilized for the follow-up assessment. In the current study, time to respond is generally extended by two seconds compared to the original task because a small pilot trial with five psychiatric patients revealed increased response times in comparison to healthy samples.

#### Statistical analysis

Demographic and clinical characteristics are only displayed descriptively as the main study (FORESEE III) is still ongoing. Difference scores from baseline to follow-up were calculated for affective empathy, compassion, and ToM separately for each group (patients with TRD and HC). Difference scores (baseline-follow-up) were then compared between groups via non-parametric Mann-Whitney U tests for two independent samples. Additionally, effect sizes “r” were calculated with r < 0.3 representing small, r < 0.5 medium and r > 0.5 strong effects [63]. We conducted non-parametric tests as the assumptions for parametric tests were not given for all variables of interest (tested using Kolmogorov-Smirnov tests for normal distribution and Levene’s test for homogeneity of variances) and considering our sample’s small size. We also ran equivalence tests [64, 65] to examine the practical similarity of affective responsiveness at follow-up between TRD patients and HC. We set the smallest effect size of interest to a large effect, with bounds of d = −0.80 (lower) and d = 0.80 (upper), and conducted a one-sided test procedure via Welch’s tests for two independent samples [66]. To analyze reliability scores, non-parametric correlation analyses of test and re-test data were calculated exclusively in the HC sample (see Supplementary Table S1). Data were analyzed using MATLAB and IBM SPSS Statistics 20. For all statistical comparisons, *p*-values ≤ 0.05 were considered significant (two-tailed).

## Results

### Sample description

As both groups were matched, gender distribution (male = 7, female = 5) and age were comparable (M_TRD_ = 44.08, SD_TRD_ = 8.08; M_HC_ = 45.33, SD_HC_ = 9.26; TRD vs. HC: U = 62, z = −0.58, *p* = 0.56, r = 0.13) (Table 1). Furthermore, both groups were comparable with regard to a measure linked to verbal intelligence, namely the multiple choice vocabulary test (MCVT) [67] (TRD: M = 112.33, SD = 14.64; HC: M = 114.67, SD = 12.77; TRD vs. HC: U = 62.5, z = −0.55, *p* = 0.58, r = 0.12). Descriptively, severity of depression assessed with MADRS, HDRS and BDI decreased in the TRD sample after the onset of DBS (MADRS: M = −10.58, SD = 9.92; HDRS: M = −8.92, SD = 8.79; BDI: M = −11.42, SD = 12.06) (Table 1). In terms of social skills, patients with TRD experienced reduced affective responsiveness (U = 26, z = −2.66, *p* = 0.01, r = 0.59) and generally reduced feelings of compassion (U = 37, z = −2.02, *p* = 0.04, r = 0.45) but intact ToM (U = 70.5, z = −0.09, *p* = 0.93, r = 0.02) at baseline compared to HC (see Supplementary Table S2). For more information about EmpaToM test data at baseline, see additional analyses in the supplement (Supplement 1).

**Table 1:** Demographic and clinical characteristics.

		Baseline	Follow-Up
TRD	N (male/female)	12 (5/7)	
	Age (mean in years) (SD)	44.08 (8.08)	
	Verbal Intelligence* (SD)	112.33 (14.64)	114.33 (14.62)
	HDRS (sum) (SD)	28.00 (4.26)	19.08 (8.89)
	MADRS (sum) (SD)	33.08 (5.96)	22.50 (10.54)
	BDI-II (sum) (SD)	38.25 (6.54)	26.83 (12.32)
HC	N (male/female)	12 (5/7)	
	Age (mean in years) (SD)	45.33 (9.26)	
	Verbal Intelligence* (SD)	114.67 (12.77)	123.33 (10.25)
	HDRS (sum) (SD)	0.83 (0.84)	0.42 (0.67)
	MADRS (sum) (SD)	0.67 (0.78)	0.42 (0.52)
	BDI-II (sum) (SD)	0.75 (1.36)	1.17 (2.37)

*TRD* Treatment-resistant depression, *HC* Healthy control subjects, *SD* Standard deviation, *HDRS* Hamilton Depression Rating Scale, *MADRS* Montgomery-Åsberg Depression Rating Scale, *BDI-II* Beck Depression Inventory II. *assessed by the Multiple Choice Vocabulary Test [67].

### Changes of social skills following DBS onset

**Fig. 2:**
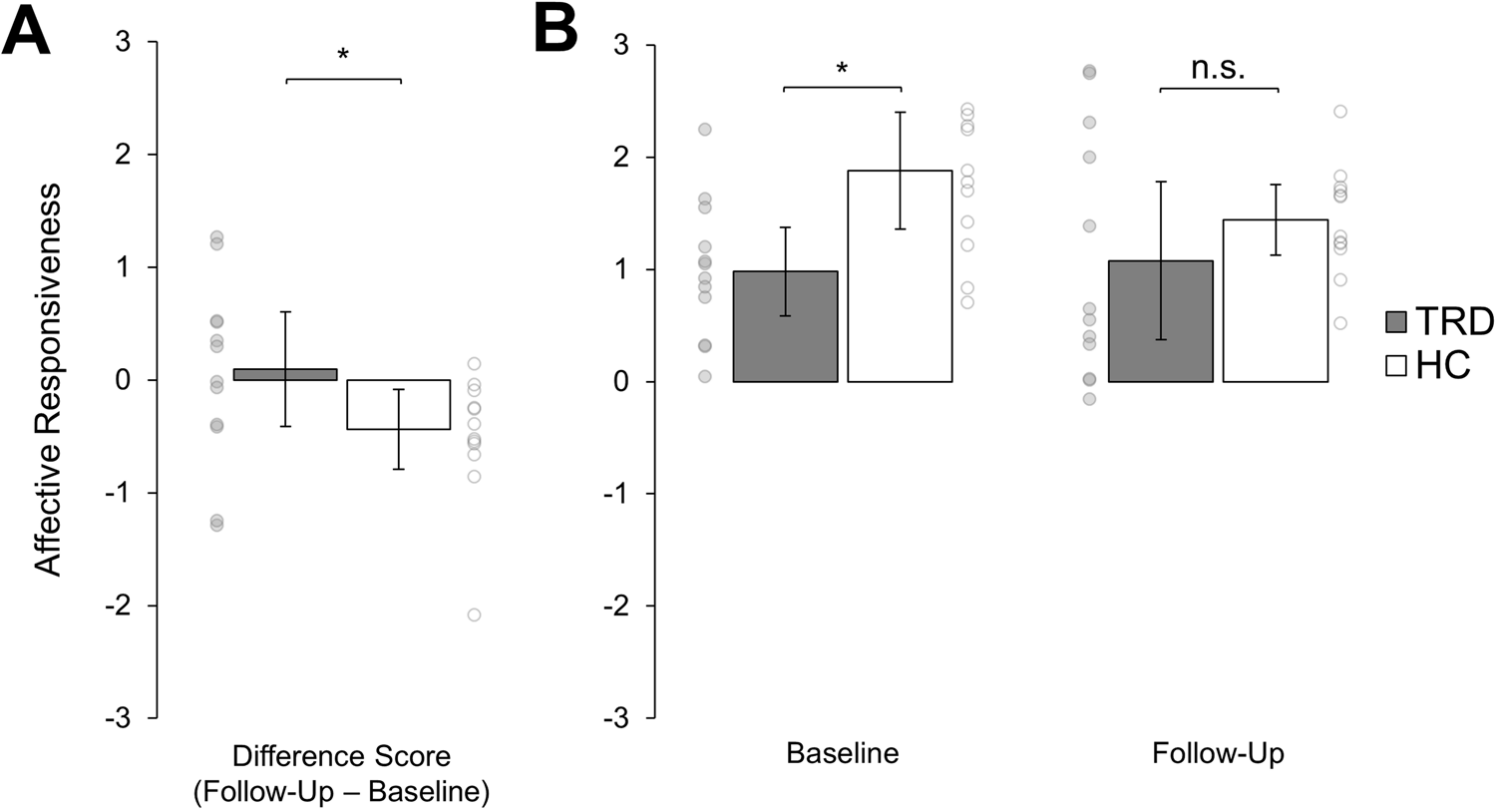
Effects on affective empathy following DBS. Shown are mean scores of differences in affect rating between negative and neutral situations (i.e. affective responsiveness) in patients with treatment-resistant depression (TRD, *n* = 12; in grey) and healthy control subjects (HC, *n* = 12; in white). Error bars represent 95% confidence intervals. Asteriks indicate a statistically significant difference (*p* ≤ 0.05, two-sided). Small dots represent individual data points. **A** Change of affective responsiveness from baseline to follow-up (difference follow-up – baseline) differed significantly between TRD patients in comparison to HC (*p* = 0.05). **B** Affective responsiveness scores separately displayed for baseline (left side) and follow-up (right side). At baseline, patients with TRD experienced significantly reduced affective responsiveness compared to HC. At follow-up no difference between the groups was found indicating a normalized affective responsiveness in patients with TRD after three months of active slMFB DBS. n.s. not significant.

To analyze the effects following three months of active slMFB DBS regarding social skills, the difference scores from baseline to follow-up assessment were compared between TRD patients (*n* = 12) and HC (*n* = 12) using non-parametric Mann-Whitney U tests (Table 2). Significant effects of medium effect size occurred in the affective empathy domain, i.e. with regard to the affective responsiveness to emotional compared to neutral stimuli (TRD: M = 0.10, SD = 0.80; HC: M = −0.44, SD = 0.56; TRD vs. HC: U = 38, z = −1.96, *p* = 0.05, r = 0.44) (see Fig. 2A). Single comparisons revealed that the affective responsiveness significantly differed between HC and patients with TRD at baseline (U = 26, z = −2.66, *p* =0.01, r = 0.59) but not at the follow-up assessement (U = 54, z = −1.04, *p* = 0.30, r = 0.23) indicating a normalized affective responsiveness (see Fig. 2B). This effect was mainly driven by changes from baseline to follow-up in the neutral condition indicating a reduction of the depression-associated negativity bias in patients with TRD (TRD: M = 0.21, SD = 0.40; HC: M = −0.18, SD = 0.32; TRD vs. HC: U = 28, z = −2.54, *p* = 0.01, r = 0.57) (Supplementary Fig. S1). To determine any equivalence in affective responsiveness at follow-up between the two groups, we conducted equivalence testing. As those results failed to reach statistical significance (T (15,17) = −1.04, *p* = 0.32), our data provide insufficient evidence to assume similar affective responsiveness at follow-up. Taken together, these findings add to the evidence of specific effects following slMFB DBS in the domain of affective empathy in terms of normalized affective responsiveness. No effects regarding other social skills (compassion, theory of mind) were revealed in the course of slMFB DBS (all *p* ≥ 0.27; for details see Table 2 and Supplementary Table S3).

**Table 2:** Group differences of changes from baseline to follow-up (difference scores follow-up - baseline).

		TRD (*n* = 12)	HC (*n* = 12)	
				Mann-Whitney U Test
		Mean (SD)	Mean (SD)	U, z, p, r
Affective Empathy	Affective responsiveness	0.10 (0.80)	−0.44 (0.56)	U = 38, z = −1.96, *p* = 0.05, r = 0.44
	Neutral	0.21 (0.40)	−0.18 (0.32)	U = 28, z = −2.54, *p* = 0.01, r = 0.57
	Emotional	−0.11 (0.52)	−0.25 (0.31)	U = 49.5, z = −1.30, *p* = 0.19, r = 0.29
Compassion	Both conditions	−0.26 (0.74)	−0.01 (0.65)	U = 63, z = −0.52, *p* = 0.60, r = 0.12
	Neutral	0.01 (0.64)	−0.02 (0.96)	U = 67.5, z = −0.26, *p* = 0.80, r = 0.06
	Emotional	−0.53 (1.13)	0.01 (0.49)	U = 53, z = −1.10, *p* = 0.27, r = 0.25
Theory of mind	Theory of Mind	0.04 (0.05)	0.02 (0.13)	U = 64, z = −0.47, *p* = 0.64, r = 0.11
	Factual Reasoning	−0.02 (0.12)	−0.05 (0.15)	U = 67.5, z = −0.26, *p* = 0.79, r = 0.06

*TRD* Treatment-resistant depression, *HC* Healthy control subjects, *SD* Standard deviation.

## Discussion

This study systematically investigated specific social skills (affective empathy, compassion, and theory of mind (ToM)) in patients with treatment-resistant depression (TRD) before and three months after the onset of deep brain stimulation (DBS) of the supero-lateral medial forebrain bundle (slMFB). Active DBS of the slMFB resulted in a normalized affective responsiveness towards emotionally negative versus neutral stimuli in patients with TRD. None of the other social skills was significantly altered following slMFB DBS. Deficits in compassion remained unchanged and socio-cognitive skills remained intact in the TRD sample.

By behaviorally assessing social skills in the course of slMFB DBS treatment using a naturalistic paradigm, this study contributes to a better understanding of DBS’s effects on social functioning. Three months following the onset of slMFB DBS (follow-up), preoperatively reduced affective responsiveness (baseline) was normalized in patients with TRD. Normalized affective responsiveness following slMFB DBS onset could represent one factor facilitating the social re-integration of these chronically ill patients [17, 20]. The increased negative affect towards neutral stimuli at baseline (e.g. depression-associated negativity bias) was significantly weaker (strong effect size) following slMFB DBS in patients with TRD compared to HC [23, 68]. This finding is in line with a previous study demonstrating a reduced negativity bias six months after the onset of DBS of the subcallosal cingulate gyrus (SCG) in nine patients with TRD [69]. Considering that the SCG and slMFB are part of the same reward-network and that the SCG is anatomically and functionally coupled with regions connected with the medial forebrain bundle [9], our data together with this previous study imply a network-specific effect of DBS in depression. Given the importance to reverse the depression-associated negativity bias for a successful antidepressant treatment [20, 70, 71], this effect might play a significant role in DBS’s antidepressant effects. Furthermore, our results appear to be promising with regard to the social skill deficits hypothesis [72], according to which persisting social skill deficits in patients with depression contribute decisively to both relapsing into depression and to chronic depressive symptoms due to the loss of positive reinforcement during social interactions. Thus, the finding of normalized affective responsiveness in the course of DBS treatment might reduce the probability of a relapse into depression and thereby contribute to a stable, long-term antidepressant effect by enabling positive social interactions. Nevertheless, considering that our equivalence tests revealed a non-significant result of affective responsiveness at follow-up, we cannot conclude that patients with TRD undergoing DBS perform as well as the HC group regarding social skills.

In contrast to affective empathy, we observed no effects following DBS on (preoperatively impaired) compassion in the TRD sample. This finding is unexpected taking into account that the slMFB is directly interconnected with the ventral striatum [41, 42], a region that has been linked to compassion [24, 73]. Although slMFB DBS is known to have rapid antidepressant effects [13, 14], the follow-up period of three months might have been too short to demonstrate effects of DBS altering compassion. Considering that affective empathy represents the basis for compassion [27, 74], feelings of compassion might only improve in the longer-term outcome subsequent to normalized affective responsiveness. Considering the crucial role of compassion in social functioning [75], it could turn out to accelerate these effects by augmenting DBS’s effects on compassion-related brain regions with specific compassion training. To date, there is no study investigating the effects of a social skills training on the antidepressant efficacy of DBS in depression. However, the value of combining DBS treatment with psychotherapy has already been established regarding other mental disorders (e.g. obsessive-compuslive disorder [76]). Therefore, it could prove worthwile to combine DBS therapy in TRD with specific trainings targeting social skill deficits. Supporting the potential of such an approach, compassion training successfully increased feelings of compassion in healthy participants accompanied by increased brain activations in the medial prefrontal cortex [77] and the ventral striatum [73].

While improvements in the domain of affective empathy on the one hand seem to be desirable and important for stable long-term outcomes and successful social functioning after recovery from depression [9, 78], potential side effects of DBS have been discussed critically in another context. Studies investigating DBS of the nucleus subthalamicus treating motor symptoms of Parkinson’s disease reported problematic behavioral changes, such as social maladjustment [79, 80] as well as worsening social skills, such as in tasks requiring emotion recognition [81] and ToM [82]. Importantly, the current study demonstrated that after three months of slMFB DBS ToM skills remained intact. This finding is comparable to a previous study in patients with TRD undergoing DBS of the subcallosal cingulate cortex [69]. Furthermore, the current data support evidence that DBS in TRD patients does not negatively alter cognition [83]. Thus, the current study has no indications for ethical concerns of slMFB DBS negatively altering social behavior.

Altogether, the key strengths of the current study are the recruitment of a unique patient sample, and the differentiated assessment of social skills following slMFB DBS by means of a naturalistic paradigm. Alongside these strengths, our study has also limitations. The patient sample of the current study is small due to the experimental status of slMFB DBS for patients with TRD in Germany. The current study thus does not allow to differentiate between DBS treatment responders and non-responders as well as to compare the effects of active and sham stimulation. Future studies are necessary to test the long-term stability of the demonstrated effects with the stimulation turned on and turned off. This is highly relevant given the fact that a discontinuation of stimulation is associated with a relapse of symptoms [16] as well as a reduction of quality of life [84].

In sum, our research demonstrated specific effects following slMFB DBS onset in depression in terms of a normalized affective responsiveness. This effect might facilitate the resumption of social activities after recovery from chronic depression thereby contributing to a stable long-term antidepressant response to DBS. Nevertheless, deficits in compassion persisted. Thus, our data support the idea to combine DBS with specific psychotherapeutic interventions for full recovery.

### Supplementary information

Supplement

Figure S1

## Data Availability

Data are available on reasonable request.
