# Supplementary material for: Normalized affective responsiveness following deep brain stimulation of the medial forebrain bundle in depression
Source: Transl Psychiatry. 2024 Jan 8;14:6. doi: 10.1038/s41398-023-02712-y (PMC10774255; doi:10.1038/s41398-023-02712-y)
Supplement: Supplementary file 1 — Supplement [file 41398_2023_2712_MOESM1_ESM.docx]

Supplement 1

**EmpaToM test parameters at baseline.**

We performed mixed ANOVAs to analyze EmpaToM test parameters at baseline. Note that in the TRD group scores of compassion condition 1 (neutral, non-ToM) (D (12) = .26, p = .023) and accuracy condition 2 (emotional, non-Tom) (D (12) = .26, p = .03) were significantly non-normal. For the compassion condition 2 (emotional, non-ToM) (F (1,22) = 13.87, p = .001) and compassion condition 4 (emotional, ToM) (F (1,22) = 5.03, p = .035) variances were significantly different in the two groups.

**Affective Empathy.** We performed a mixed ANOVA with between-subjects factor “group” (HC, TRD patients) and two within-subjects factors “valence” (neutral, emotional) and “task” (ToM; non-ToM). We found a significant main effect for “valence” (F (1,22) = 92.75, p < .001, η² = .81). Furthermore, interaction effects of “valence” and “group” (F (1,22) = 9.12, p = .006, η² = .29) as well as “valence” and “task” (F (1,22) = 6.63, p = .017, η² = .23) revealed significant results. These results demonstrate, that the EmpaToM test parameters were similar compared to previously reported data (Kanske et al., 2015).

**Compassion.** We performed a mixed ANOVA with between-subjects factor “group” (HC, TRD patients) and two within-subjects factors “valence” (neutral, emotional) and “task” (ToM; non-ToM). We found a significant main effect for “valence” (F (1,22) = 149.08, p < .001, η² = .87) and for “group” (F (1,22) = 4.69, p = .041, η² = .18) as well as a significant interaction effect of “valence” and “task” (F (1,22) = 11.49, p = .003, η² = .34).

**Theory of Mind.** We performed a mixed ANOVA with between-subjects factor “group” (HC, TRD patients) and two within-subjects factors “valence” (neutral, emotional) and “task” (ToM; non-ToM). We found a significant main effect for “task” (F (1,22) = 8.76, p = .007, η² = .29) and a significant interaction effect for “task” and “group” (F (1,22) = 5.597, p = .027, η² = .20).

Taken together these results demonstrate the same results as previously published in Kilian et al., (2022). These results also demonstrate that the EmpaToM results are similar to the validation study (Kanske et al., 2015).

Supplement 2

Table S1

| Non-parametric correlation coefficients of test and re-test scores in HC. | | |
| --- | --- | --- |
|  |  | HC (n = 12)  r, p |
| Affective Empathy | Affective responsiveness | r = .88, p < .001 |
|  | Neutral | r = .63, p = .028 |
|  | Emotional | r = .65, p = .022 |
| Compassion | Both conditions | r = .68, p = .015 |
|  | Neutral | r = .71, p = .009 |
|  | Emotional | r = .62, p = .031 |
| Theory of mind | Theory of Mind | r = .47, p = .122 |
|  | Factual Reasoning | r = .46, p = .135 |
| *Note.* HC = healthy control subjects. | | |

Supplement 2

Table S2

| Group differences in affective empathy, compassion and theory of mind at baseline. | | | | |
| --- | --- | --- | --- | --- |
|  |  | TRD (n = 12) | HC (n = 12) |  |
|  |  |  |  | Mann Whitney U Test |
|  |  | Mean (SD) | Mean (SD) | U, z, p, r |
| Affective Empathy | Affective responsiveness | 0.98 (0.62) | 1.88 (0.82) | U = 26, z = -2.66, p = .01, r = .59 |
|  | Neutral | -0.13 (0.34) | 0.54 (0.36) | U = 3, z = -3.98, p < .001, r = .89 |
|  | Emotional | -1.11 (0.68) | -1.34 (0.56) | U = 57, z = -0.87, p = .39, r = .19 |
| Compassion | Both conditions | 2.44 (1.25) | 3.38 (0.83) | U = 37, z = -2.02, p = .04, r = .45 |
|  | Neutral | 1.37 (1.19) | 2.37 (1.05) | U = 36, z = -2.08, p = .04, r = .46 |
|  | Emotional | 3.50 (1.48) | 4.38 (0.69) | U = 48, z = -1.39, p = .17, r = .31 |
| Theory of mind | Theory of Mind | 0.66 (0.13) | 0.66 (0.13) | U = 70.5, z = -0.09, p = .93, r = .02 |
|  | Factual Reasoning | 0.51 (0.11) | 0.64 (0.15) | U = 34.5, z = -2.17, p = .03, r = .49 |
| *Note.* TRD = treatment-resistant depression, HC = healthy control subjects, SD = standard deviation. | | | | |

Supplement 2

Table S3

| Socio-affective and socio-cognitive skills at baseline and follow-up (mean values). | | | |
| --- | --- | --- | --- |
|  |  | Baseline | Follow-Up |
|  |  | Mean (SD) | Mean (SD) |
| TRD  (n = 12) | Affective responsiveness | 0.98 (0.62) | 1.08 (1.11) |
|  | Neutral | -0.13 (0.34) | 0.08 (0.25) |
|  | Emotional | -1.11 (0.68) | -0.99 (0.94) |
|  | Compassion (both conditions) | 2.44 (1.25) | 2.18 (1.59) |
|  | Neutral | 1.37 (1.19) | 1.38 (1.41) |
|  | Emotional | 3.50 (1.48) | 2.97 (1.94) |
|  | Theory of Mind | 0.66 (0.13) | 0.69 (0.11) |
|  | Factual Reasoning | 0.51 (0.12) | 0.49 (0.13) |
| HC  (n = 12) | Affective responsiveness | 1.88 (0.82) | 1.44 (0.49) |
|  | Neutral | 0.54 (0.36) | 0.35 (0.28) |
|  | Emotional | -1.34 (0.56) | -1.09 (0.41) |
|  | Compassion (both conditions) | 3.38 (0.83) | 3.37 (0.76) |
|  | Neutral | 2.37 (1.05) | 2.35 (1.19) |
|  | Emotional | 4.38 (0.69) | 4.39 (0.54) |
|  | Theory of Mind | 0.66 (0.13) | 0.68 (0.12) |
|  | Factual Reasoning | 0.64 (0.15) | 0.59 (0.16) |
| *Note.* TRD = treatment-resistant depression, HC = healthy control subjects, SD = standard deviation. | | | |

Supplement 3

Figure S1. DBS effects on affective empathy. Shown are mean scores of affect ratings for each condition (neutral and emotional) in patients with treatment-resistant depression (TRD, n = 12; in grey) and healthy control subjects (HC, n = 12; in white) for each time point (baseline and follow-up). Error bars represent 95% confidence intervals. Asteriks indicate a statistically significant difference (p ≤ .05, two-sided). Small dots represent individual data points.
