# Supplementary figures and images for: Normalized affective responsiveness following deep brain stimulation of the medial forebrain bundle in depression
Source: Transl Psychiatry. 2024 Jan 8;14:6. doi: 10.1038/s41398-023-02712-y (PMC10774255; doi:10.1038/s41398-023-02712-y)

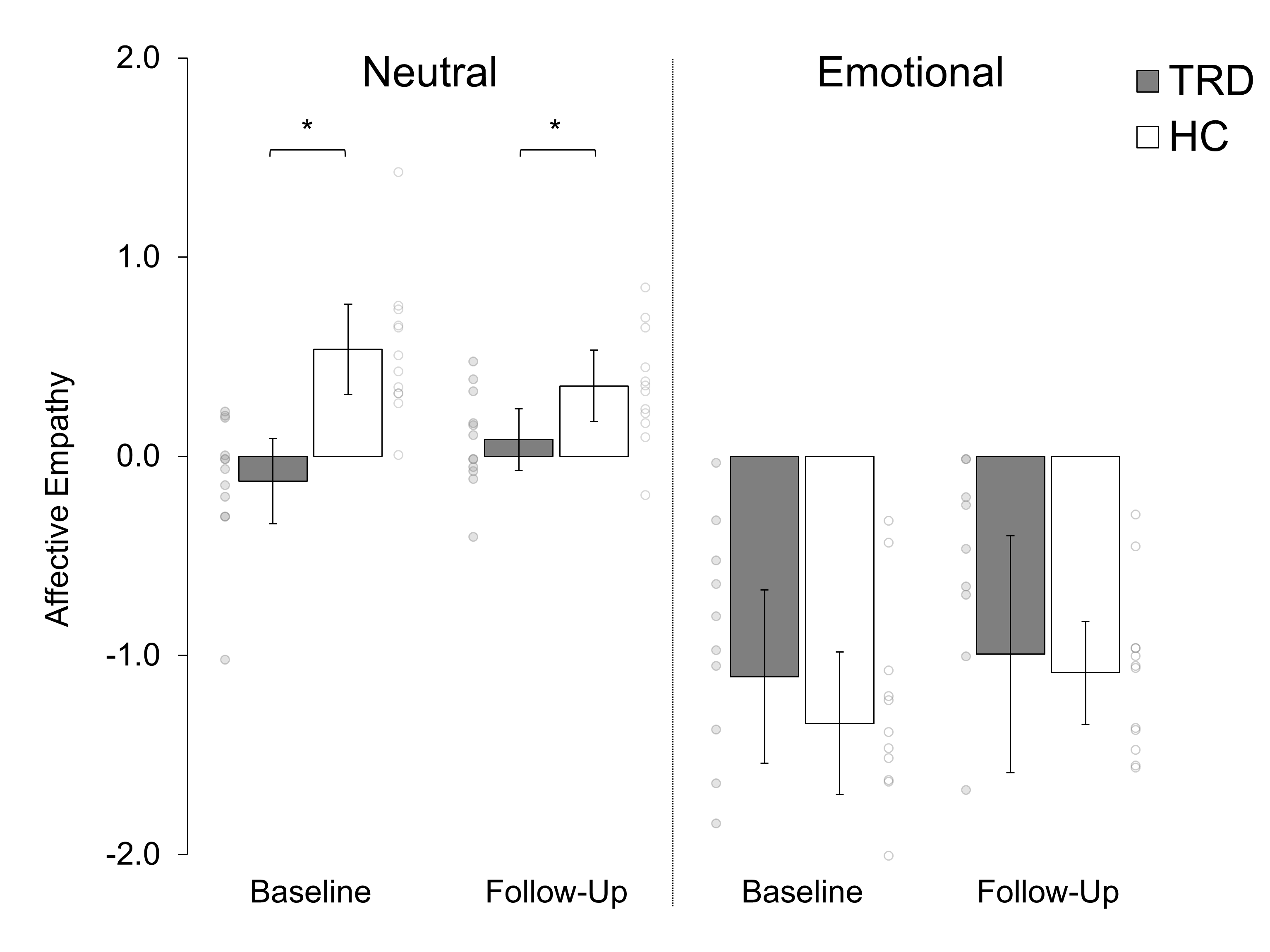

Supplement: Supplementary file 2 — Figure S1 [file 41398_2023_2712_MOESM2_ESM.tif]
